# A Feasibility Study into the Use of Three-Dimensional Printer Modelling in Acetabular Fracture Surgery

**DOI:** 10.1155/2015/617046

**Published:** 2015-01-29

**Authors:** A. W. Yu, J. M. Duncan, J. S. Daurka, A. Lewis, J. Cobb

**Affiliations:** ^1^St Mary's Hospital, London W2 1NY, UK; ^2^Orthopaedic Department, Charing Cross Hospital, London W6 8RF, UK; ^3^Imperial College NHS Trust, London SW7 2AZ, UK

## Abstract

There are a number of challenges associated with the operative treatment of acetabular fractures. The approach used is often extensive, while operative time and perioperative blood loss can also be significant. With the proliferation of 3D printer technology, we present a fast and economical way to aid the operative planning of complex fractures. We used augmented stereoscopic 3D CT reconstructions to allow for an appreciation of the normal 3D anatomy of the pelvis on the fractured side and to use the models for subsequent intraoperative contouring of pelvic reconstruction plates. This leads to a reduction in the associated soft tissue trauma, reduced intraoperative time and blood loss, minimal handling of the plate, and reduced fluoroscopic screening times. We feel that the use of this technology to customize implants, plates, and the operative procedure to a patient's unique anatomy can only lead to improved outcomes.

## 1. Background

Operative management of acetabular fractures is often highly complex and presents to the surgeon with a number of challenges. These include the choice of approach, adequate visualization of relevant anatomy, reduction techniques, and method of fracture fixation. Frequently, the surgical approach used is extensive [1], while operative time and perioperative blood loss can often be significant [2], all of which have been shown to be important prognostic indicators of surgical outcome [3, 4]. Currently, two- and three-dimensional computed tomography (CT) images are used in conjunction with plain X-rays to aid spatial understanding of complex fracture patterns. However, given their intangible nature, they provide only limited insight into the three-dimensional configuration of fracture components and thus the optimal surgical management [5, 6]. Successful fixation often requires advanced spatial awareness and multiple intraoperative direct fluoroscopic images, which can result in increased intraoperative time and radiological exposure.

Additionally, the ilioinguinal approach described by Letournel and Judet [1] offers safe anatomical windows to allow for fracture manipulation and fixation. However, whist working in these limited windows, it can often be difficult to appreciate the three-dimensional anatomy of the anterior column and its relationship to the posterior column, the quadrilateral surface and the hip joint. In particular in situations where there has been previous lower abdominal surgery, it is often more difficult and dangerous to develop sufficiently sized windows to allow for adequate visualization of the surrounding anatomy. This can lead to an extension in the operative time, increased blood loss, and a higher risk of infection.

The advent of 3D printing technology, a method of rapid manufacturing with additive scintillation, has been termed by some as the “third industrial revolution” [4]. Numerous fields from aerospace to civil engineering have already embraced this technology to produce inexpensive, fast, and precise models for various ends. We feel that the availability of an economical and rapid method of generating patient specific and highly detailed models of complex fractures as well as recreating preinjury anatomical morphology has important implications in acetabular fracture surgery. Not only can it aid preoperative planning of surgical fixation and reduction but it can also allow for real-time, intraoperative appreciation of individualised skeletal morphology as well as complex acetabular anatomical relationships.

## 2. Methods

**Figure 1:**
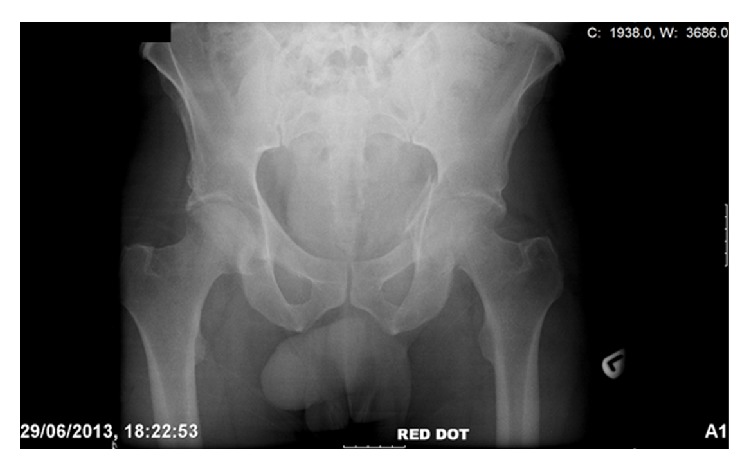
An AP radiograph taken at the time of initial presentation revealing a left acetabular fracture.

**Figure 2:**
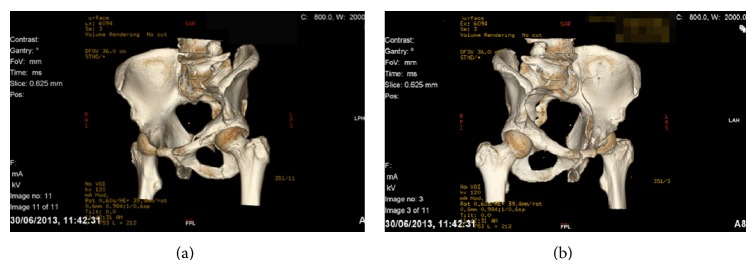
(a) An obturator oblique CT reconstruction. (b) An iliac oblique CT reconstruction.

**Figure 3:**
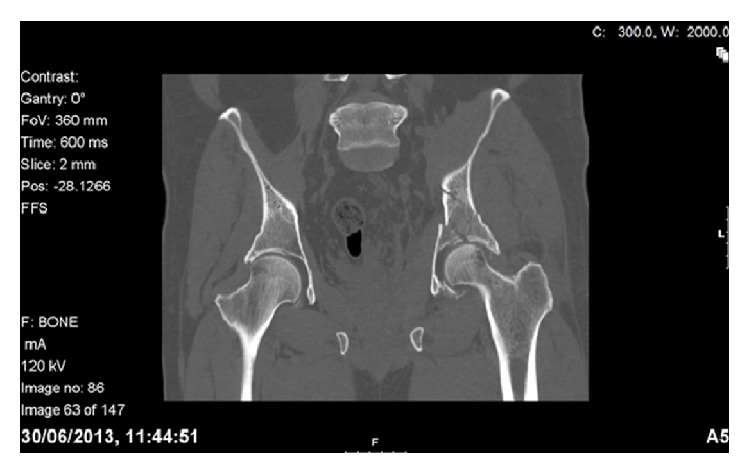
A coronal CT reconstruction suggesting medial subluxation of the femoral head.

**Figure 4:**
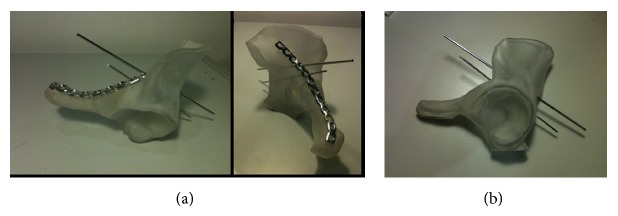
(a) 3D printed model with contoured plate applied. (b) 3D printed model with posterior column trajectories demonstrated.

In 2013, we identified two patients who presented to our level one major trauma centre with both column fractures of the left acetabulum. In both cases there was associated quadrilateral plate and posterior column displacement allowing for joint subluxation. Patient 1 was a forty-eight-year-old male who sustained the injury from a motorcycle accident in the Sahara Desert. Patient 2 was a fifty-six-year-old diabetic patient who presented after a mechanical fall whilst attempting to jump off a boat. Both had comminuted fractures of the left acetabulum as isolated injuries that were visualised on X-ray and CT (Figures 1, 2(a), 2(b), 3, 4(a), and 4(b)). The CT scans taken at the time of presentation were taken with conventional 16-detector CT scanners and included 0.625 mm axial sections that were used to reconstruct a 3D model of the pelvis. The uninjured right hemipelvis of the model was reflected and reformatted to model an uninjured left hemipelvis. Two trajectories were superimposed on this model to simulate screws from a pelvic brim plate that could lag the posterior column fragment, avoiding displacement of the posterior wall fragment and any intra-articular penetration. The DICOM imaging files were converted to STL to enable printing on an Objet Eden 250 printer utilising selective laser sintering of Objet MED610 polymer (Stratasys Inc., Rehovot, Israel). The printing process depended on the size of the desired output. Thus in order to minimize the printing time to less than 8 hours, we only printed what was strictly required. Our model exhibited the mirror image of the uninjured hemipelvis and extended from the sacroiliac joint to the pubic symphysis (Figure 4(a)).

## 3. Results

Preoperatively, the model allowed visualisation and tactile assessment of the multiple fracture fragments and their relative displacements. The relative positions of the quadrilateral plate and posterior column fragments could be assessed and the surgeon felt that these would be amenable to reduction from an ilioinguinal approach. The confidence with which this could be decided preoperatively prevented the patient from undergoing a dual or more extensive approach.

Intraoperatively, the 3D model presented the two screw trajectories as 2 mm diameter circular defects in the model to allow 2 mm K-wires to be inserted. This aided visualization and tactile memory of the most appropriate plate hole to use as well as the angle required for optimum positioning. The 3D printed model included the pelvic brim from the pubic symphysis to the sacroiliac joint and caudally the limit of printing was to the most inferior point of the acetabulum, including its attached posterior wall and column (Figures 4(a) and 4(b)).

**Figure 5:**
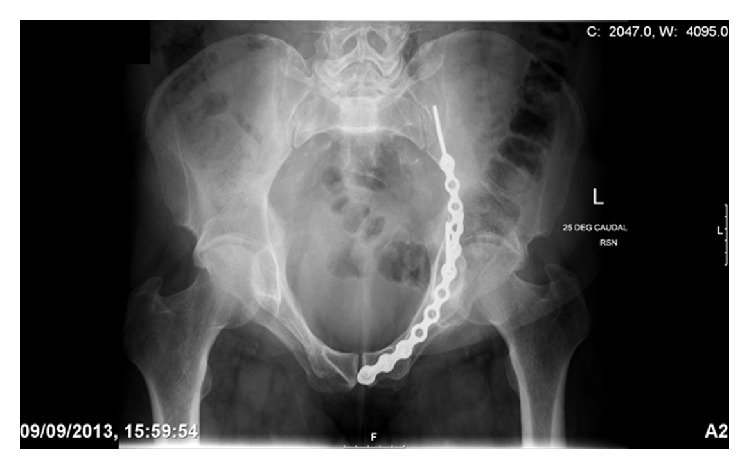
An inlet view of the pelvis to highlight the plate contouring achieved.

In the supine position, the left leg was draped for traction to be applied by a surgical assistant. All 3 windows were developed and used. The intraoperative tactic involved identification of the anterior column fracture and removal of any loose bone fragments preventing reduction. Development of the middle window allowed visualisation of the quadrilateral plate and posterior column fragments. The medial subluxation and the anterior column fragments were reduced with use of traction and a Matta offset pelvic clamp to hold the reduction. This was temporarily augmented with 2 mm threaded wires. Concurrently an assistant used the 3D model to size a pelvic reconstruction plate (Synthes Inc., West Chester, USA) to place on the pelvic brim and contoured it appropriately. The plate was introduced onto to the anterior column via the medial window and moved into position using all 3 windows. No further contouring was required to enable it to sit on the pelvic brim appropriately; screws were inserted to hold the anterior column reduction. The posterior column/quadrilateral plate fragment was held using the previously planned screw trajectory on the 3D model (Figure 5) to provide absolute stability of fracture reduction. Fluoroscopic views were obtained to ensure that reduction was anatomical and there was no evidence of intra-articular screw penetration. The procedures took on average 146 minutes, with an average estimated blood loss of 450 mls. Use of a red cell saver in patient 2 allowed recovery and infusion of 162 mls of the patient's own blood.

## 4. Discussion

There are previous examples of 3D models being used in orthopaedic surgery through the use of rapid prototyping [6–8]. However, this method has so far been limited due to its expensive and time-consuming nature. With the proliferation of additive processes, user-friendly software, and the use of CT scanning in the early assessment of injuries, 3D printing technology offers a number of advantages to surgeons treating complex fractures. It can allow for assessment of the injury type and improved appreciation of the fracture fragments in order to aid planning.

In our study it augmented the stereoscopic 3D CT reconstructions, allowing an understanding of the normal 3D anatomy of the pelvic brim, acetabular columns, and their relationship to the hip joint. This information was used intraoperatively to select and contour an appropriate pelvic reconstruction plate with 6 degrees of freedom. After the reduction of the fracture had been achieved, the plate was applied with no further adjustments. The advantages for the patients include reduction in the associated soft tissue trauma of the surgical procedure due to a minimisation of tissue handling and mobilization. Both accurate precontouring of the plate and limited surgical dissection contributed to this. The minimal handling of the plate and single contouring attempt prevented repeated recontouring which can have a negative effect on its mechanical properties [8]. Fluoroscopic imaging was also minimized as the inserted metalwork could be seen to be appropriate on the initial views, which did not require adjustment. Use of a 3D model led to reductions in operative time, minimisation of blood loss, and a reduced risk of infection.

With the increasing economy and speed of additive processing, the applications of 3D printing in orthopaedic surgery are far reaching. As a technology its merits are yet to be fully explored. For example, there are already reports of direct laser metal sintering to produce custom titanium miniplates in maxillofacial surgery [9]. The use of 3D modeling, custom made implants, and plates unique for a patient's anatomy and fracture type seems to offer further advantages and is a prospect that we feel can only lead to improved outcomes. We feel that further research into this growing field is necessary not only to explore further avenues where this technology can be applied but also to further develop the auxiliary technologies for sterilization of 3D printed models and its safety profile for use in surgery. We eagerly anticipate further research and developments in the use of 3D printing in orthopaedics.
